# Case of Traumatic Occlusion of the Vagina

**Published:** 1858-05

**Authors:** Ernst Schmidt


THE CHICAGO

MEDICAL JOURNAL.

VOL. I. MAY, 1858. No. 5.

### ORIGINAL COMMUNICATIONS.

### ARTICLE I.

### CASE OF TRAUMATIC OCCLUSION OF THE VAGINA.

(Read before the Cook County Medical Society, at its regular meeting, April, 1858.)

BY ERNST SCHMIDT, M.D.

The complete occlusion of the vagina, if acquired by any traumatical or pathological influences in the time of sexual maturity, belongs as well to the rarest as to the most interesting diseases and deformations of the sexual system of women; because, it firstly arrests at once all functions of the whole series of highly-important organs, and, secondly, always results fatally without operative help and attempting the latter in most cases. Therefore, I think that the following case which I successfully treated, with my friend, Dr. W. Wagner, in this city lately, may be worthy to claim your attention for a few minutes:

I begin with the history, as far as it could be gathered from the statement of the patient and her friends. She is a small, slender woman, of a very lively and somewhat nervous temperament, twenty-three years of age, and cannot remember any former sickness worth mentioning. Her monthly terms first appeared in her sixteenth year, and returned almost every fortnight till she got married in her twentieth, without any bad effect from this irregularity upon the state of her health; since which time the courses came on pretty regularly every fourth week, and her whole constitution improved. Having conceived two years after her marriage, and feeling quite comfortable during the whole term of pregnancy, she had the first labor at the right time, on the 25th of July, 1857. The pains at first were very strong and frequent, by and by decreased in number and strength, and two days having elapsed, the water was not yet broken. The attending midwife at last burst it, at a time when the pains had almost totally disappeared. No further pains coming on, the midwife continued to manipulate for twelve hours more with her hands in the vagina and upon the belly, and ordered the woman in labor to press down as much as her sinking energies allowed.

Under this rational treatment, the head by and by was pressed into the entrance of the vagina, so that, as the bystanders state, it already was visible. At the same time, the mother asserts, she still felt very violent movements of the child in her abdomen. But as now no further advancement could be attained, the husband at last ran for a doctor, and finally found one, that is to say, he found a fellow, who once served as a barber in Germany, but at present has a splendid sign parading at his office door in South Wells street, * * * "Physician, Surgeon and Accoucheur." This illustrious sample of the medical profession then made his appearance, and for some hours manipulated on the child's scalp with his finger-nails, and towards evening of the 28th July, succeeded in delivering a still-born, well developed child, weighing twelve pounds, and as the husband reported, "quite dead," and the face and scalp horribly scratched and skinned; he is also sure, that the child's face looked backwards, so that it was no face presentation. Then the so-called doctor pulled the navel-cord in such a manner, that it was torn, and having obtained this glorious result, as well as his ten dollar fee, he left the house so well satisfied, that he did not care any further for the course of the confinement, as I suppose, the most thankworthy feature for the wretched mother. Half an hour later, the returning midwife found only the navel-cord, but not the placenta, and this could only be brought to-day after hard working for another half hour.

Notwithstanding all this ill-treatment, the loss of blood after the delivery was trifling. However, the patient became very weak, had some chills alternating with heat, complained of considerable soreness in the abdomen and diarrhœa, and lost from the several organs a great quantity of bad smelling matter for about a fortnight. The urine always passed without difficulty. On the twelfth day of her confinement, suddenly a considerable quantity of blood flowed out of genitals, and at the same time both legs showed a large œdematous swelling, which again disappeared after a fortnight. At this period, the patient commenced leaving her bed, and after a while also the suppuration from the genitals and the diarrhœa stopped, and so after the lapse of six weeks from confinement, the patient considered herself fully recovered.

But she never regained her former good health and strength, but fell away by degrees. Two weeks later, violent pains appeared in the back and in the region of the ovaries, and generally all the symptoms peculiar to menstrual-colic and retention of the menses by mechanical impediment. These pains lasted in their highest intensity from two to four days, and from thence returned in regular intervals from three to four weeks, more and more increasing in intensity. The intervals were rather free, but the husband soon discovered that the normal size of his wife's vagina was shortened and contracted in a very offending manner. Possibly, this critical circumstance assisted in calling in medical aid; at least, it was recommenced towards the end of October, during one of the violent attacks coincident with the period of her terms. The treatment comprehending the strongest internal and external emmenagoga, without any previous manual examination, was followed up by a great number of druggists, midwives and quacks, till the 19th of January, 1858. Thus, the attacks increased in violence and duration, and at last the intervals between them almost disappeared entirely. The patient, worn out by pains, continued sleeplessness, constipation, and impossibility to take sufficient food, could hardly be prevented from destroying her own life.

She threw herself to the floor tremendously, crying with convulsive fits. Just after such a scene, I saw her the first time, on the 10th of January, 1858.

As all the reported symptoms must seem to be caused by a retention of the menses, a manual exploration was immediately resorted to. The abdomen, especially in its right side, looked swollen; palpation established exactly corresponding to the region of the right ovarium; a spherical tumor, about double as large as a man's fist, from which a cord two fingers thick could be traced almost to the midst of the abdomen towards the symphysis. The tumor could be dislocated to and fro without difficulty; the percussion sounds over it were quite dull. The left side is free, with normal tympanitic sound; likewise just over the symphysis, neither by a deep pressure, nor by percussion, is any tumor to be traced. The attempt to make an exploration through the vagina shows at once the immediate cause, as the finger could not be introduced above the labia minora more than an inch. The first finger-joint completely fills the whole of this rudimentary vagina, nor can any aperture leading farther up be found by the touch. Inspection shows the opening of the urethra a little pressed down; the commissura posterior shows hard cicatrices running backwards to the anus, in front and to the inside of the vagina, which apparently originate from several very considerable lacerations of the perinæum. A very small glass speculum introduced between the labia pudendi showed the bottom of the vagina completely closed, as also manifold explorations by probes of all kinds, made it evident, that no communication existed with the uterus. The membrane of the vagina had lost the character of a mucous membrane, and was dry and without the specific secretion. The internal manual exploration being very painful, and the callous, scarred state of the upper end of the rudimentary vagina making the exploration more difficult, it was impossible to settle, whether there existed above the closing cicatrized cord a residue of the former regular upper part of the vagina, tightly filled with blood, or whether the uterus was in immediate contact. The finger exploring through the rectum at once hardly an inch above the anus, touched a spherical tumor as big as the fist, that could be nothing but the reflexed and distended fundus uteri, and that when moved a little communicated the movement distinctly to the tumor of the right ovarium and *vice versa.* This result of the exploration of course hardly allowed any doubts for the diagnosis, nor for the prognosis; for the intervening cicatrix being so very strong and tough, the natural secretions and excretions of the several organs, together with those heaped up already, could not be expected to force their exit, and that, therefore, the patient's life probably very soon would be destroyed if surgical aid were not resorted to; the more so, as not only the patient by the most distressing pains was already almost exhausted, but also the danger was near at hand, that the distended ovarium or the tube should burst and be followed by a fatal peritonitis. Therefore, Dr. Wagner and I at once determined to undertake the operation without delay, on the 12th of January. The patient was then laid across her bed in a position similar to that for the operation of lithotomy and then anæsthetised. Dr. Wagner stood at her left side, the index finger of his left hand in the anus, and with the right hand holding a silver catheter lying in the bladder. I knelt down between her legs, brought the index of my left hand into the vagina like a probe, upon which I introduced a very fine-pointed and somewhat-crooked tenotome with the edge upon the concave side. The attempt always to feel with my index finger whether the knife is not approaching either Dr. Wagner's finger in the anus or the catheter, may theoretically easily be recommended, but in practice it is a good deal more difficult, especially in a case where the rudiment of the vagina was so very contracted. In an equal distance from rectum and the catheter, I penetrated the vagina, and pressed the knife rigorously forward about half an inch deep, till I felt that the resistance was yielding; then I dilated the opening externally by the incision about half an inch long, whereupon thick tar-like blood commenced flowing out, and in this way indicated that the first success was attained. In the meantime, the patient began to manifest great debility. The pulse becoming imperceptible, respiration was more accelerated, and only the first mitral sound was to be heard distinctly and almost always coincident with a deep respiration, a sign which we should be always mindful of, as it reminds us immediately to cease the anæsthetic, even when the state of the pulse does not yet seem to require it. By the application of ether, cold water, etc., we soon succeeded in checking the effect of chloroform, and the patient was removed to her usual bed-position. The dark blood continued to flow away slowly and sparingly, perhaps six ounces in the first two hours.

Then, at once, in the afternoon, from one to three o'clock, about two to three pounds of blood of a very offensive smell, mixed with black and quite solid coagula, was expelled under moderate pains. At five o'clock P.M., pulse ninety-six beats, small, irregular; the ovarium tumor in the right side had become smaller and was already sunken down into the pelvis, so that it only could be felt by deep pressure. The fundus uteri palpable through the rectum equally had decreased in size and now stood fully two inches above the anus entrance. Evidently the blood oozing during the first two hours had come from a space between the rudimentary vagina and the uterus; when this space was emptied the fully retroverted uterus was able to rise again; the orificium uteri, during the rising of the organ, withdrew from the symphysis and the bladder against which it had been pressed in the former pathological position; and in this way, now two hours after the operation, the uterus, the tubes, and the ovaria, began to empty themselves. The following day a piece of compressed sponge was introduced into the incision. The patient feels comfortably. The third day, no further flowing, and suddenly all the symptoms of a circumscribed but rather acute peritonitis arose. The sponge was instantly removed. Pains dnring the night almost unbearable, pulse small (100), towards evening, 108 beats. A little blood oozing out again at noon. The 15th of January, in the morning, pulse 148, nose pointed, cheek red, cold sweats, cool extremities, the abdomen to the navel very tender and painful when touched; only a little purulent, bad smelling fluid from the vagina; in the evening some more flow, pulse down to 132 beats, stronger. Under the proper external and internal treatment, the symptoms of the peritonitis, so dangerous for the patient's life, by and by abated, after three days, though the tenderness of the abdomen and vagina, the costiveness and the fever, made attentive care necessary for another week. At this time also the purulent flux stopped, and pure blood oozed till the 24th of January. Then the tumor in the high ovarium region had totally disappeared, nor was any tumor felt through the rectum. An elastic catheter can easily be introduced through the incision in the cervical canal and uterus, which therefore has assumed a very favorable position. The well known glairy secretion of the cervical canal is always emptied by this manipulation. However, only a small space seems to exist between the present vagina and the cervix uteri. The introduction of a catheter in this way was continued from four to six weeks, about every other day, till the fear could be abandoned that the incision might unite again. Meanwhile the menses have returned three times regularly in intervals of three weeks. The woman is now enjoying good health and strength as before her confinement. The vagina is widened and quite a finger long, certainly a striking contrast to that state in which we had found it. The incision well cicatrized in its circumference has almost entirely disappeared, but allows a two line cathether to enter into the cervical canal without difficulty. Thus the cure may be considered a complete one; and one may perhaps only regret, that the constriction has not been opened and widened so far as to lodge in it the cervix uteri as in another fundus vaginæ, and to cause a fastening of the cervix with the surface of the incision by adhesive inflammation. However, the next success is the best; the more so, as it is very doubtful whether the experiment to try the latter operation would not have been followed by a rapidly fatal peritonitis—the latter having been dangerous enough in our method of limited operation. But a more difficult and important question, I suppose, arises, what will be done in case of another conception—a matter worthy perhaps a further discussion?

